# Eicosapentaenoic Acid and Oxypurinol in the Treatment of Muscle Wasting in a Mouse Model of Cancer Cachexia

**DOI:** 10.1371/journal.pone.0045900

**Published:** 2012-09-20

**Authors:** Vanessa C. Vaughan, Melanie Sullivan-Gunn, Edward Hinch, Peter Martin, Paul A. Lewandowski

**Affiliations:** 1 School of Medicine, Deakin University, Waurn Ponds, Australia; 2 School of Biomedical and Health Sciences, Victoria University, Melbourne, Australia; University of Minnesota Medical School, United States of America

## Abstract

Cancer cachexia is a wasting condition, driven by systemic inflammation and oxidative stress. This study investigated eicosapentaenoic acid (EPA) in combination with oxypurinol as a treatment in a mouse model of cancer cachexia. Mice with cancer cachexia were randomized into 4 treatment groups (EPA (0.4 g/kg/day), oxypurinol (1 mmol/L ad-lib), combination, or control), and euthanized after 29 days. Analysis of oxidative damage to DNA, mRNA analysis of pro-oxidant, antioxidant and proteolytic pathway components, along with enzyme activity of pro- and antioxidants were completed on gastrocnemius muscle. The control group displayed earlier onset of tumor compared to EPA and oxypurinol groups (P<0.001). The EPA group maintained body weight for an extended duration (20 days) compared to the oxypurinol (5 days) and combination (8 days) groups (P<0.05). EPA (18.2±3.2 pg/ml) and combination (18.4±3.7 pg/ml) groups had significantly higher 8-OH-dG levels than the control group (12.9±1.4 pg/ml, P≤0.05) indicating increased oxidative damage to DNA. mRNA levels of GPx1, MURF1 and MAFbx were higher following EPA treatment compared to control (P≤0.05). Whereas oxypurinol was associated with higher GPx1, MnSOD, CAT, XDH, MURF1, MAFbx and UbB mRNA compared to control (P≤0.05). Activity of total SOD was higher in the oxypurinol group (32.2±1.5 U/ml) compared to control (27.0±1.3 U/ml, P<0.01), GPx activity was lower in the EPA group (8.76±2.0 U/ml) compared to control (14.0±1.9 U/ml, P<0.05), and catalase activity was lower in the combination group (14.4±2.8 U/ml) compared to control (20.9±2.0 U/ml, P<0.01). There was no change in XO activity. The increased rate of weight decline in mice treated with oxypurinol indicates that XO may play a protective role during the progression of cancer cachexia, and its inhibition is detrimental to outcomes. In combination with EPA, there was little significant improvement from control, indicating oxypurinol is unlikely to be a viable treatment compound in cancer cachexia.

## Introduction

Many forms of cancer present with a complex metabolic profile characterized by loss of lean body mass and adipose tissue, known as cancer cachexia. Approximately half of all cancer patients develop cachexia [1], with the prevalence rising as high as 86% in the last 1–2 weeks of life [2], and 20% of cancer deaths attributable to cachexia [3]. Patients suffering cachexia may lose up to 30% of their original body weight, with 45% of patients losing more than 10% of their original weight over disease progression [4].

Oxidative stress may play an integral role in cancer cachexia, with evidence of oxidative damage and high levels of reactive oxygen species (ROS) found in many cancer states [5]. The role of ROS in the development of cancer cachexia, and the mechanisms that cause the disease remain largely unknown, despite several advances in identifying circulatory factors and pathways that are active. A shift in the balance between reactive oxidants and antioxidants can induce a state of oxidative stress that is detrimental to the cell, and may be one of the key factors in the development of cachexia.

Superoxide dismutase (SOD) is an enzyme responsible for the dismutation of superoxide anion (O_2_^−^·) into hydrogen peroxide and oxygen. The activity of SOD has been shown to be decreased in the cachectic state [6], [7], indicating that there is an inability to compensate for increases in ROS, and therefore an inability to protect the cell from oxidative stress in the cachectic state. The antioxidant enzymes catalase and Glutathione Peroxidase (GPx) that break down hydrogen peroxide into oxygen and water have also been found to have lower activity in cancer cachexia studies [8], indicating that the systems could be key contributors to oxidative stress and damage observed in cancer cachexia.

Whilst some of the pathways involved in the excess production of ROS have been studied at length in cancer cachexia, there are others that have been shown to play a role in oxidative insult in other diseases, which have yet to be studied in detail in cancer cachexia. Xanthine oxidoreductase is an enzyme with two distinct forms that are responsible for catalyzing the conversion of hypoxanthine to xanthine, and xanthine to uric acid [9]. Xanthine Dehydrogenase (XDH) is expressed *in vivo*, and uses NAD+ as an electron acceptor for the reduction reaction, forming NADH. In the presence of pro-inflammatory mediators, XDH readily cleaves into Xanthine Oxidase (XO), which instead uses molecular oxygen for the conversion of hypoxanthine to xanthine, and xanthine to uric acid, producing the highly reactive O_2_^−^·, or hydrogen peroxide [9].

**Figure 1:**
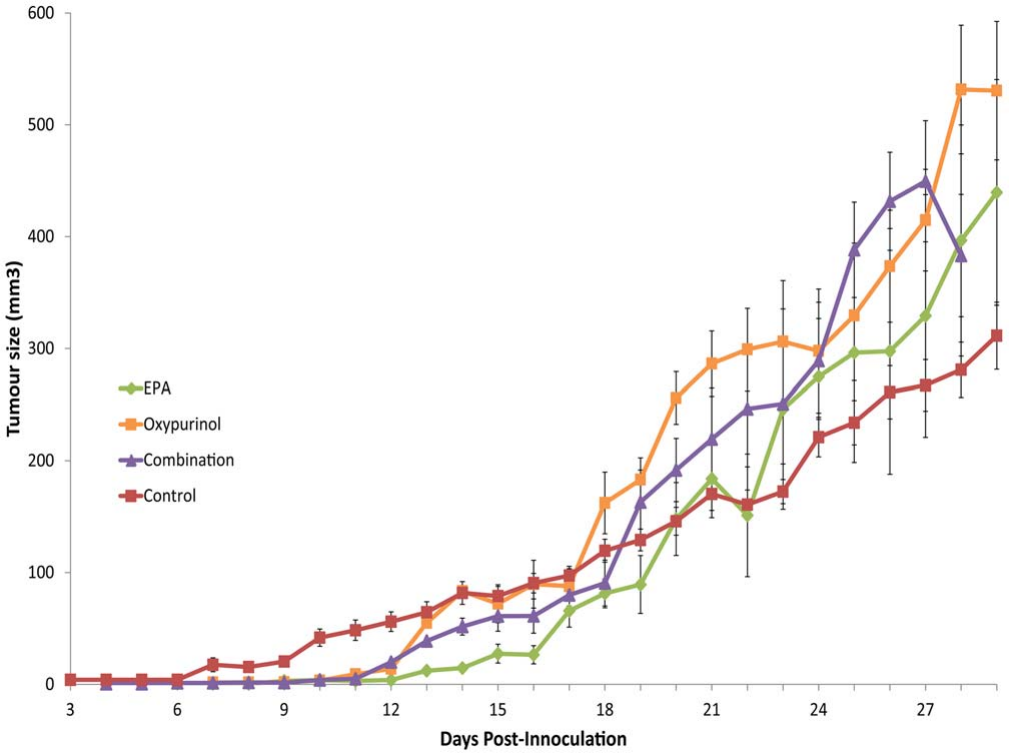
Tumor growth. Data presented as mean ± SEM.

Whilst XO is not usually present at high levels in skeletal muscle, raised levels are commonly seen in muscle tissue damage and ischemia-reperfusion injury [10]. High levels of XO have also been observed in the blood of some cancer patients compared to patients without cancer [11], and it is been suggested that cachectic animals respond favorably when treated with XO inhibitors [12]. The abundance of pro-inflammatory factors present in cachexia may lead to an increase in the cleavage of XDH to the XO form, explaining higher circulating levels of XO. Increased levels of XO would then lead to the excess production of ROS, and contribute to oxidative stress in cancer cachexia.

**Figure 2:**
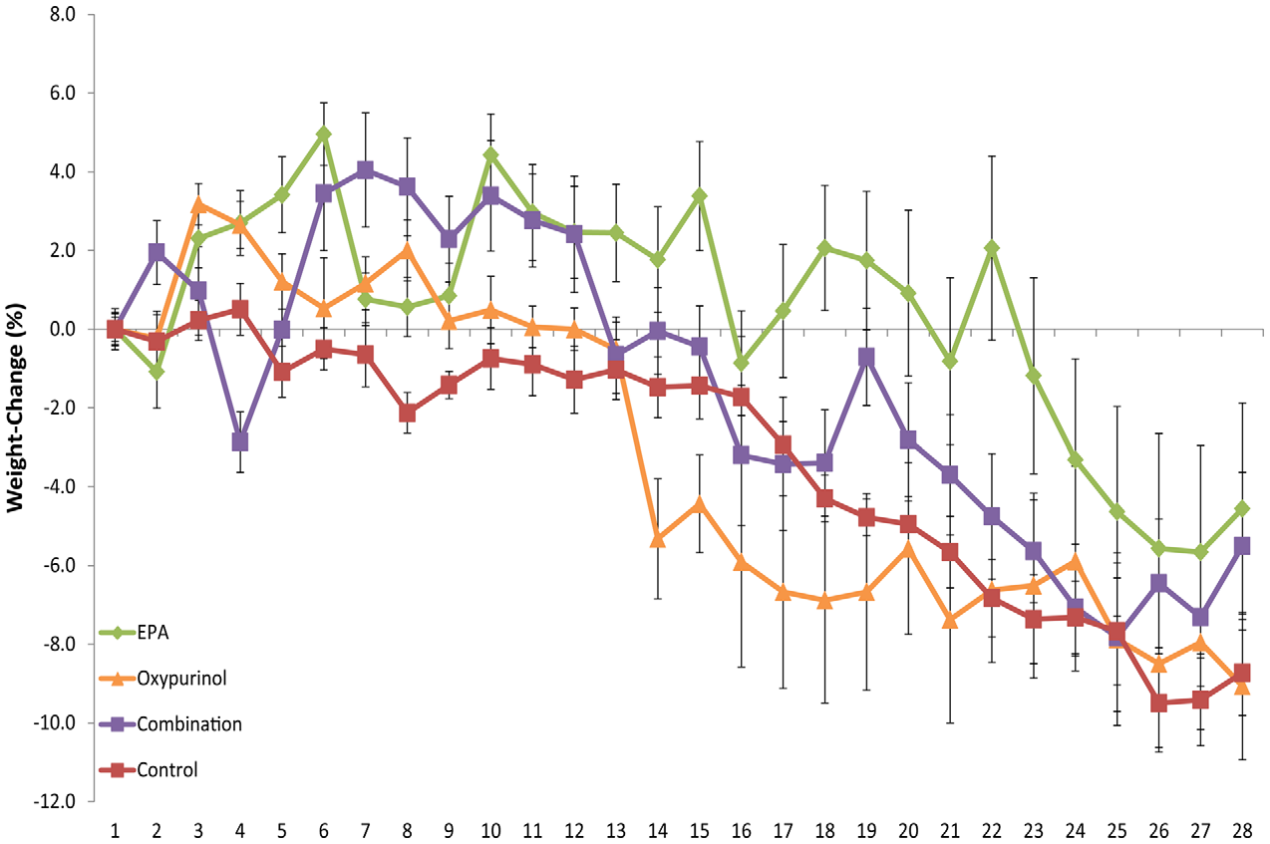
Weight-loss over time. Data presented as mean ± SEM.

Oxypurinol is a noncompetitive, irreversible inhibitor of XO, considered more potent than allopurinol, of which it is a metabolite [10]. Currently oxypurinol is used as a treatment for conditions in which XO is a contributor, and has been shown to decrease tissue wasting and increase cardiac function in cachectic animals [12], [13]. A decrease in purine production and associated metabolism requirements may prompt the reduction of XDH expression, and therefore downstream activity of this enzyme. Uric acid, produced by XO, increases the conversion of arachidonic acid into its biologically active metabolites [14]. This in turn increases the activation of NADPH Oxidase, perpetuating the signaling cascade that results in the activation of increased transcription of the components of the ubiquitin-proteasome system.

**Figure 3:**
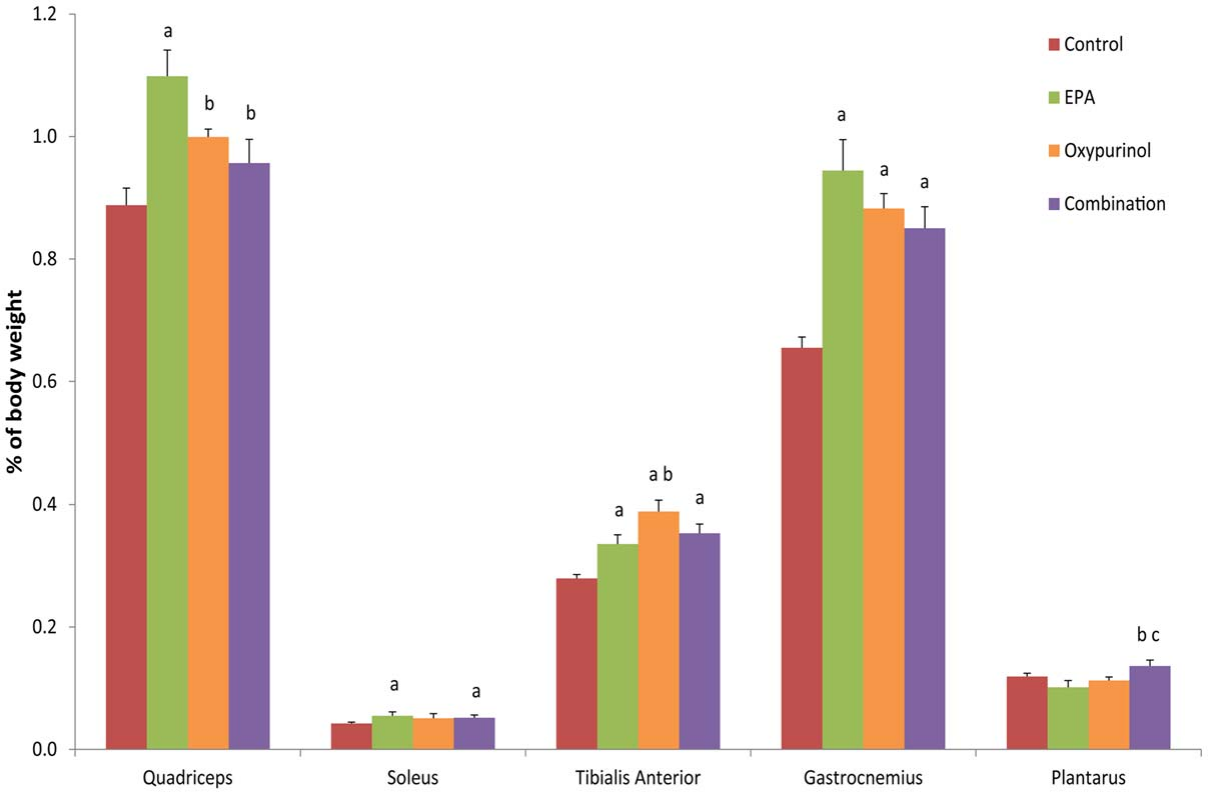
Wet muscle weights as percentage of final body weight. Data presented as mean ± SEM. ^a^ significantly different compared to control, ^b^ significantly different compared to EPA, ^c^ significantly different compared to oxypurinol (P<0.05).

The progressive catabolism of muscle in cancer cachexia suggests a pivotal role in systems of protein degradation, such as the ubiquitin proteolytic pathway (UPP). The UPP has been found to be up-regulated both in experimental models and patients with cachexia [15], [16], indicating contribution to muscle loss and associated negative outcomes. Before proteins are degraded by the UPP, they must be targeted by conjugated to multiple molecules of ubiquitin. In order for this conjugation to occur, ubiquitin must first be activated by an ubiquitin-activating enzyme (E1), and then transferred to the active site of an ubiquitin carrier protein (E2). The bound E2 recognizes ubiquitin conjugating enzymes (E3 or E3 protein ligase), which allow conjugation reactions to take place, which form a chain of ubiquitins linked to each other and the protein substrate. Only when ubiquitin is targeted to a selected protein can it then be recognized by the proteasome, and processed into smaller peptides [17]. Several E3 protein ligases have been shown to be active during proteolysis in muscle atrophy, in particular muscle-specific F-box (MAFbx)/atrogin-1 and muscle specific ring finger 1 (MURF-1) [18].

**Figure 4:**
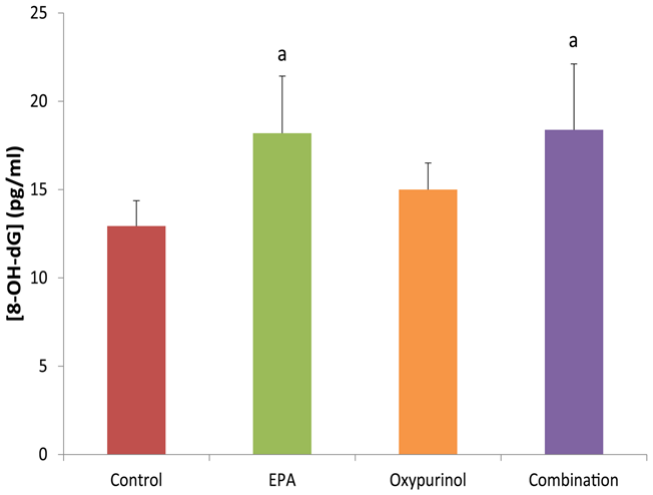
Gastrocnemius 8-OH-dG levels as a marker of oxidative stress. Data presented as mean ± SEM. ^a^ significantly different compared to control, ^b^ significantly different compared to EPA, ^c^ significantly different compared to oxypurinol (P<0.05).

Eicosapentaenoic acid (EPA) is a naturally occurring omega-3 fatty acid, found in oily fish and certain algae, widely considered to have great potential as an antigenotoxic, antioxidant and chemopreventive agent. Administration of EPA has been shown to increase the activity of the ROS scavenging SOD [19], slow the development of some cancers, and increase weight gain and quality of life in pancreatic cancer patients [20]. In recent years, EPA has been trialed as a treatment for cancer cachexia due to its various roles as an agonist of SOD and in upstream regulation of the expression and activity of the UPP [20]–[22]. EPA replaces arachidonic acid in phospholipid membranes when consumed at high levels [23], and has also been shown to inhibit 15-HETE production from arachidonic acid, which has been implicated in UPP regulation in murine models of cachexia [24], [25]. Some animal trials of EPA have been successful, as has its combination with other therapeutic approaches in human patients, such as leucine supplementation, high protein diet and exercise [26], [27].

**Table 1:** Gene expression in gastrocnemius muscle.

Gene	EPA	Oxypurinol	Combination
**CuZnSOD**	0.90±0.1	1.28±0.2	0.80±0.1^c^
**MnSOD**	1.69±0.1	1.86±0.25^a^	0.93±0.2^c^
**EcSOD**	1.36±0.3	1.02±0.1	0.29±0.3^abc^
**GPx1**	3.08±0.3^a^	4.14±0.3^a^	1.38±0.3^bc^
**CAT**	1.80±0.4	2.39±0.2^ab^	0.58±0.2^c^
**XDH**	1.02±0.5	2.69±0.3^ab^	0.66±0.3^c^
**NOX2**	1.03±0.2	0.61±0.2	0.53±0.2^b^
**MAFbx**	2.51±0.4^a^	3.56±0.3^a^	0.65±0.2^bc^
**MURF-1**	3.21±0.3^a^	2.76±0.4^a^	0.51±0.2^bc^
**UbB**	1.17±0.3	1.30±0.2^a^	0.73±0.2^c^

Data presented as fold change relative to control. ^a^ significantly different compared to control, ^b^ significantly different compared to EPA, ^c^ significantly different compared to oxypurinol (P<0.05).

The current study aimed to establish whether inhibition of xanthine oxidase by oxypurinol had a beneficial effect in treatment of muscle wasting in cancer cachexia. Further, the researchers sought to ascertain whether EPA in combination with oxypurinol was an effective multimodal treatment for muscle wasting in cancer cachexia. It was hypothesized that the inhibitory effect of oxypurinol on XO combined with the agonistic effect of EPA on SOD would reduce the presence of excess O_2_^−^·in cachectic muscle, leading to a reduction in oxidative insult and resultant injury or wasting when compared with cachectic controls.

**Figure 5:**
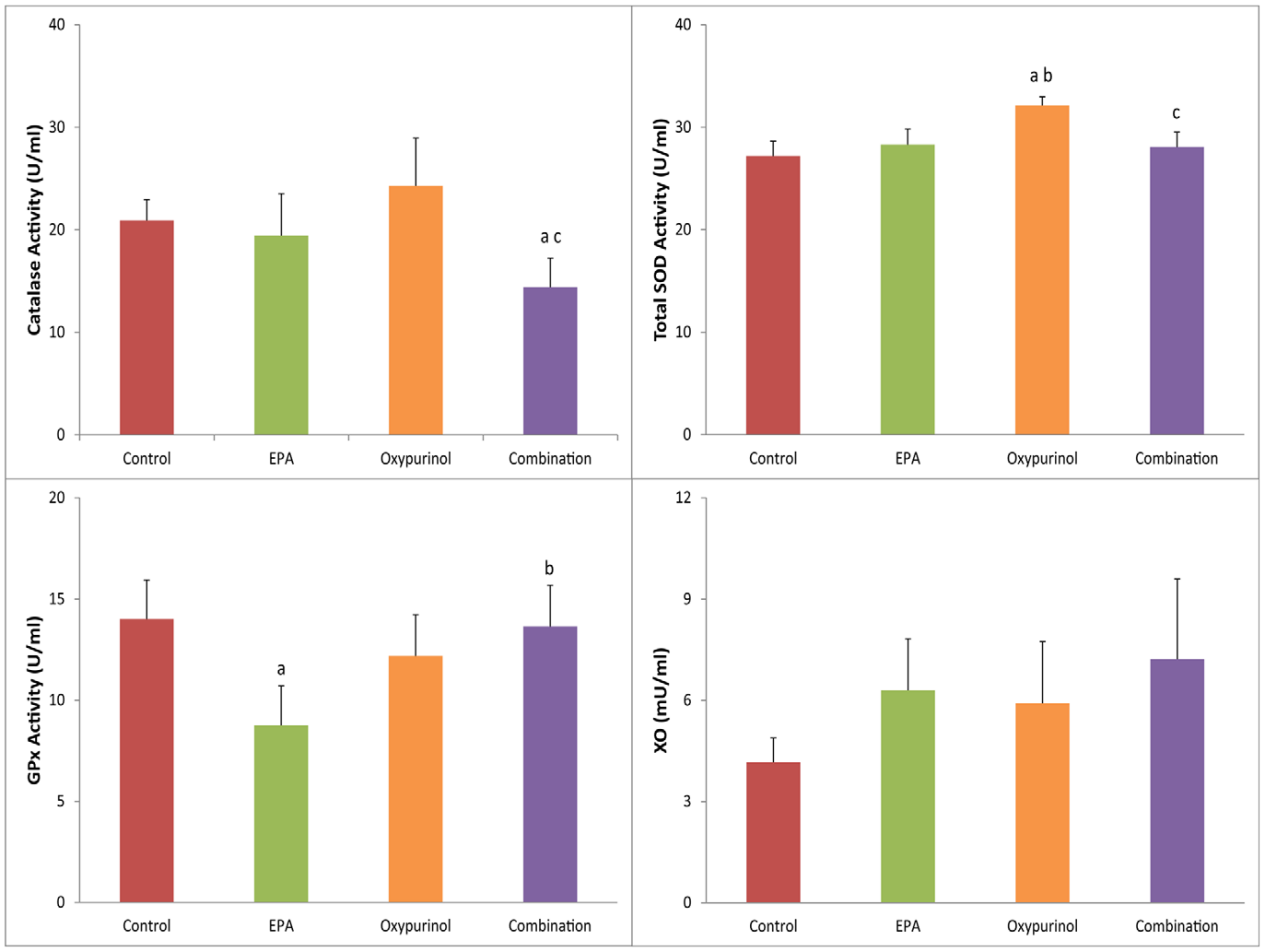
Enzyme activity in gastrocnemius tissue. Data presented as mean ± SEM. ^a^ significantly different compared to control, ^b^ significantly different compared to EPA, ^c^ significantly different compared to oxypurinol (P<0.05).

**Table 2:** PCR primers for gene expression.

	Forward	Reverse
CuZnSOD	5′-TGAACCAGTTGTGTTGTCAG-3′	5′-TCCATCACTGGTCACTAGCC-3′
EcSOD	5′-AGGTGGATGCTGCCGAGAT-3′	5′-TCCAGACTGAAATAGGCCTCAAG-3′
MnSOD	5′-TGGCTTGGCTTCAATAAGGA-3′	5′-AAGGTAGTAAGCGTGCTCCCACAC-3′
CAT	5′-CGGCACATGAATGGCTATGGATC-3′	5′-CGGCACATGAATGGCTATGGATC-3′
GPx1	5′-GGGCTCCCTGCGGGGCAAGGT-3′	5′-ATGTACTTGGGGTCGGTCATG-3′
XDH	5′-ATCTGGAGACCCACTGCACC-3′	5′-TGTGCTCACGAAGAGCTCCAT-3′
NOX2	5′-TTGGGTCAGCACTGGCTCTG-3′	5′-TGGCGGTGTGCAGTGCTATC-3′
Ubiquitinβ	5′-GCTCAGTGACGAGAGGCTTT-3′	5′-TCACGAAGATCTGCATTTTGA-3′
MAFbx	5′-AAGATCAAACGCTTGCGAAT-3′	5′-GAACATCATGCAGAGGCTGA-3′
MURF1	5′-AGGAGCAAGTAGGCACCTCA-3′	5′-GTCCATGTCTGGAGGTCGTT-3′

## Results

### Animal Model

Four animals from the control group and one each from the oxypurinol and combination treatment groups were culled prior to the conclusion of the trial due to ethical concerns, and were not included in statistical analyses.

Mice in the control group showed tumor onset from 6±0.3 days (Figure 1), whilst the age and weight matched EPA (10±0.7 days) and oxypurinol (9±0.6 days) treatment groups had a significant delay in tumor onset by comparison (P<0.001). The EPA group also had significantly delayed onset compared to the combination group (7±0.7 days, P<0.001). The oxypurinol treatment delayed tumor onset compared to the combination (P<0.01) group. There was no statistical difference in size of final tumor between EPA (338±67 mm^3^) and control (308±28 mm^3^) groups, while oxypurinol (489±78 mm^3^) and combination (515±41 mm^3^) treatment groups showed increased final tumor size compared to both control (P<0.01) and EPA groups (P<0.05).

Weight-loss was calculated as a percentage of initial weight, corrected for tumor mass (Figure 2). The control group experienced significant weight-loss compared to initial weight from day 12 onward (P<0.05). The EPA group increased in body weight during the pre-cachectic phase, before weight stabilization, and maintained this pre-cachectic state until day 22, when weights started to decline, with significant weight-loss from day 25. The oxypurinol group experienced gradual decline from peak weight from day 3, before a sharp decrease in weight at day 13. Weight loss was significant in this group from day 14 and gradually decreased to endpoint. The combination group recovered from a sharp decline in body weight at day 4, significantly increasing in body weight from initial, and were stable before experiencing significant weight-loss from day 17 (excluding day 19; P<0.01). The control group had average weight change of −9.06% at euthanasia, the EPA group −4.91%, oxypurinol −9.11% and the combination group −5.03%, There was no significant differences in percentage weight-loss between groups at euthanasia. The EPA treatment group displayed weight-loss significantly lower than the control group (P<0.05) for an extended duration (Days 3–15, 17–23, total 20 days) compared to both the oxypurinol (Days 3, 4, 7, 8, 10, total 5 days) and combination (Days 6–12, 19, total 8 days) treatment groups. Whilst there was a very strong correlation between tumor size and weight loss in the control group (r^2^ = 0.94), less variation was explained by the correlation between weight loss and tumor size in the EPA (r^2^ = 0.72), oxypurinol (r^2^ = 0.72), and combination (r^2^ = 0.70) treatment groups.

In the EPA treatment group, quadriceps, soleus, TA and gastrocnemius muscles constituted a significantly higher percentage of total body weight than the control group at end point (P≤0.05; Figure 3). In the oxypurinol treatment group, TA and gastrocnemius were also a significantly higher percentage of total body weight compared to the control group at end point (P≤0.05). Weights of soleus, TA and gastrocnemius were also greater than the control group in animals subjected to the combination treatment (P≤0.05). Quadricep weight increased in the EPA treatment group compared to both oxypurinol and combination treatments, however TA and plantarus were also decreased compared to oxypurinol and combination groups, respectively.

### Oxidative Stress

8-OH-dG levels in gastrocnemius muscle from the control group was 12.9±1.4 pg/ml. EPA (18.2±3.2 pg/ml) and combination (18.4±3.7 pg/ml) groups had significantly higher levels than the control group (P≤0.05; Figure 4), indicating increased oxidative stress in these groups. The oxypurinol group did not display significantly higher 8-OH-dG levels compared to control (14.9±1.5 pg/ml, P≤0.07).

### Gene Expression

Gene expression in gastrocnemius muscle is shown in Table 1. The antioxidant component GPx1 increased 3.1-fold in the EPA group and 4.1-fold in the oxypurinol group compared to control (P<0.01), as did the expression of the ubiquitin E3-ligases MURF1 and MAFbx, >2.5-fold in the EPA group, and 2.8-fold and 3.6-fold in the oxypurinol group respectively (P<0.05). Oxypurinol also increased expression of antioxidant components MnSOD by 1.9-fold (P<0.05) and CAT by 2.4-fold (P<0.01) compared to control, while XDH increased 2.7-fold (P<0.01) and a 1.3-fold increase in proteasome subunit UbB (P<0.05) was observed. The treatments in combination decrease EcSOD expression 0.3-fold compared to control values (P<0.05).

### Enzyme Assays

Activity of total SOD significantly increased in the oxypurinol group (32.2±1.5 U/ml; Figure 5) compared to control (27.0±1.3 U/ml, P<0.01), combination (25.0±3.4 U/ml, P<0.01) and EPA groups (28.3±1.5 U/ml, P<0.05). There was no change between other groups. Activity of GPx was significantly reduced in the EPA group (8.76±2.0 U/ml) compared to both the control (14.0±1.9 U/ml, P<0.05) and combination groups (13.6±2.0 U/ml, P<0.05). Catalase activity was reduced in the combination group (14.4±2.8 U/ml) compared to both the control (20.9±2.0 U/ml, P<0.01) and oxypurinol groups (24.3±4.7 U/ml, P<0.05). There were no other significant changes between groups. There was no significant difference in XO activity between groups.

## Discussion

EPA has been generally accepted to have the potential to assist in the treatment of cancer cachexia, in particular as part of a combined approach to therapy [26], [27], however further elucidation of the exact mechanisms is required. In the current study, animals treated with EPA had significantly delayed tumor development, and therefore improved outcomes, which may have been due to EPA's previously described anti-tumor action [28], rather than anti-cachectic action alone. However, while inhibited tumor growth may partially explain attenuation of weight loss, previous research indicates that the anti-cachectic effect of EPA is larger than would be expected given the magnitude of tumor reduction [29], and appears to elicit a response distinct from the anti-tumor effect [30], supporting the view that the increased variation seen in the EPA group in this study may be due to factors other than inhibited tumor growth. Future studies would benefit from the animals being culled at weight-loss or tumor size limits, rather than arbitrary dates, in order to establish if animals treated with EPA experience similar weight-loss to untreated animals with similar tumor size and mass, despite delayed onset.

Long chain n-3 PUFA, such as EPA, are known to be compromised by oxidative stress, forming lipid hydroperoxidases and fatty acid peroxyl radicals which in turn may damage lipid membranes [31]. The incorporation of EPA into lipid membranes of cells in animals undergoing supplementation [23] may increase the susceptibility of these membranes to oxidative damage, including cell and nuclear membranes, and further exposing cell contents including DNA, to the increased oxidative state, and may explain the increased oxidative damage to DNA indicated by the levels of 8-OH-dG in the EPA group. 8-OH-dG is the predominant form of free radical-induced oxidative lesion, and is commonly used as biomarker for oxidative stress [32]. 8-OH-dG is produced during oxidative damage to DNA, caused by interaction between the hydroxyl radical and nucleotide bases of DNA strands, and is considered a well characterized and sensitive marker of such damage [32].

EPA treatment caused a decrease in GPx activity compared to the control group at end point. This decrease in antioxidant capacity may also be indicative of a decreased requirement for antioxidant action, due to either reduced ROS presence or increased ROS scavenging by other enzymes. There was also no change in SOD or catalase activity. Together, this indicates that EPA's action is not as an agonist of antioxidant activity as hypothesized, and that by end point, the potential for increased antioxidant action indicated by the increase in GPx1 gene expression is being dampened in this group. Thus despite changes in gene expression that in the absence of disease may have caused increased activity of antioxidant enzymes such as GPx, the presence of cancer cachexia prevented such functional changes from occurring. By completion of the study, the EPA group had begun to decline in weight, and it is therefore possible that an earlier effect of EPA on antioxidant function caused the delayed onset of weight-loss; however further time-course studies are required to confirm this hypothesis. The decline in body weight in the EPA group at the end of the study was indicative of cancer cachexia being present.

A previous study has indicated that inhibition of XO by oxypurinol treatment reduced loss of total body weight and lean body mass, and reduced the production of ROS compared to controls [12]. However, the current study suggests that oxypurinol treatment may cause adverse outcomes in the rodent model, in particular the rapid decline of performance indicated by weight-loss seen early in disease. Up-regulation of components of the UPP also suggests a shift toward muscle wasting in this group. Indeed, oxypurinol, rather than decreasing the activity of XO, caused no overall change when compared to the control group. When seen in context of the increased gene expression of XDH, this indicates that there may be an inhibitory effect, which is being compensated for by increased production of XOR. The oxypurinol treatment group also exhibited a trend toward increased oxidative damage compared to control (<0.07) indicating there may be an increased abundance of ROS. Increased activity of SOD in this group compared to control shows increased scavenging of ROS, most likely in response to this increase in oxidative damage. These differences in response may be due to a low bioavailability of oxypurinol in the method of administration used in this study [13], and alternative, higher potency preparations or modes of delivery should be considered in future studies. Future studies into the role of XOR and purine metabolism in cachexia may also benefit from inhibition of this pathway at a point upstream of XOR. However, due to differences between rodent and primate metabolism of purines, it is important that this pathway be investigated fully in a model that allows clinical parallels to be drawn.

During study progression, the combination group displayed weight-loss phase characteristics similar to both the EPA and oxypurinol groups. For example, the initial weight increase and stabilization reflects the EPA group, the sharp decline at 12 days mirrors that of the oxypurinol group at 14 days. The amount of weight lost by the combination group is, for the most part, not as extreme as the oxypurinol group, but weight is not as well preserved as the EPA group. These weight-loss patterns are also distinct from the control group, indicating that it is the combination of the two groups, rather than a lack of effect, causing this pattern of decline. An interaction between the two treatments is further supported by gene expression and enzyme activity data, where the effect in the combination group is significantly different from the effect of either treatment alone.

Given the significant changes in many parameters experienced in the combination therapy group compared to either component in isolation, it is possible that the pathways altered by EPA and oxypurinol are linked. Although EPA has been previously shown to suppress urate crystal-induced inflammation in Sprague-Dawley rats [33], this has not previously been shown in a model of cachexia. Further research is required, but the intersection of these two pathways at arachidonic acid is the most likely point of interaction [14], [23], [24]. The combined inhibition of the UPP by EPA and oxypurinol is supported by the reduction in gene expression of UbB, MURF1, and MAFbx in this study (See Table 1). Inhibited action of the pathway upstream of NADPH Oxidase by EPA and oxypurinol is further supported by the trend toward a decrease in expression of NOX2 in the combination treatment group compared to control (P<0.06), and the decreased expression of NOX2 in the combination group compared to the EPA group indicates that the compound effect of the combination therapy is increasing inhibition of this pathway, most likely via the previously proposed mechanism. A decrease in purine production and associated metabolism requirements, triggered by feedback inhibition of amidophosphoribosyl transferase [13], may prompt the reduction of XDH expression seen in the combination group. The significant changes seen in the combination group may also be caused by increased uptake of oxypurinol due to altered permeability of cell membranes caused by EPA supplementation, and may be the cause of the apparent interaction. Altered fluidity and phospholipid composition of cell membranes has been observed following n-3 PUFA supplementation [34], modifying the uptake of hydrophobic drugs. Given the relatively high lipid solubility of oxypurinol [13], this alteration would allow the drug to pass easily via passive diffusion.

### Conclusion

EPA continues to show promise as part of a multi-modal treatment for cachexia, with a positive shift towards maintenance of lean muscle mass, and delayed onset of weight-loss in animals undergoing EPA treatment compared to those receiving other treatments. Mechanisms of protection were no longer active at end-point studied, therefore time-course studies are required to determine those responsible for the protective effects observed. Oxypurinol appears to cause an increased rate of weight decline in mice with cancer cachexia compared to all other treatment groups. This indicates that XO may play a protective role during the progression of cancer cachexia, and its inhibition is detrimental to outcomes. However, preservation of mass in certain muscles implies that oxypurinol may be beneficial in maintaining muscle mass. The increased effects in the combination group compared to the EPA or oxypurinol treatments suggest an intersection of the two mechanistic pathways, or that there in an increased uptake of oxypurinol in the presence of EPA supplementation, causing the apparent interaction. Further investigations are required to fully elucidate the interactive effect of oxypurinol and EPA, and the role of XO in cancer cachexia.

## Materials and Methods

### Cell Culture

The murine adenocarcinoma 16 (MAC16) cell line was cultured in RPMI with 10% FBS and 0.5% penicillin/streptomycin (Invitrogen, Mulgrave, Australia). Cells were grown to 80% confluence, centrifuged at 500 *g* for 5 minutes at 4°C, and isolated from the growth media. Cells were then resuspended in sterile PBS, and drawn into a 25-gauge needle for injection.

### Animal Model

All animal experiments carried out in this study were approved by the Animal Welfare Committee, at Deakin University (approval number A13/2010). Female Balb/c *nu nu* mice aged 8 weeks (Animal Resource Centre, Canning Vale, Australia) were housed in groups of 5, with free access to standard chow and water throughout the study, weighed weekly to determine consumption. Ambient temperature was controlled at 22°C±2°C, at 40–60% humidity, with a 12-hour light/dark cycle. Mice were injected with previously prepared cells, then randomized into 4 groups, consisting of EPA treatment (EPAX, Aalesund, Norway, 0.4 g/kg/day, n = 9 [EPA]), oxypurinol treatment (Sigma-Aldritch, Castle-Hill, Australia; 1 mmol/L in drinking water, n = 9), combination treatment (as per EPA and oxypurinol groups n = 9), or the cancer cachexia control group (n = 13). Animals were monitored daily for changes in body weight, and tumor sizes measured using calipers. Mice were terminated by sodium pentobarbital injection (30 mg/kg) (Virbac Animal Health, Regents Park, Australia) at 29 days, when weight loss reached 25%, or tumor size reached 1000 mm^3^, whichever occurred first. Muscle tissues, including gastrocnemius, soleus, plantarus, tibialis anterior (TA), and quadriceps, were removed and weighed. All samples were snap frozen and stored at −80^o^C for further analysis.

### Oxidative Stress

An ELISA kit was used to measure the DNA oxidation byproduct 8-hydroxy-2-deoxy guanosine (8-OH-2dG) (StressMarq Biosciences, Victoria, Canada) as a marker of oxidative stress [32]. DNA was extracted from 10 mg of gastrocnemius muscle using a DNA isolation kit (Promega, Sydney, Australia). Each sample was then diluted so that 50 µg of DNA was used in the 8-OH-2dG assay. The competitive immunoassay involves the binding of free 8-OH-2dG to an antibody coated 96 well plate. The assay and sample concentration of 8-OH-2dG were carried out as per the manufacturer's instructions.

### Gene Expression

Total RNA was extracted from 10 mg of frozen gastrocnemius muscle using TRI reagent (Astral Scientific, Sydney, Australia) according to the manufacturer's specification. The total RNA concentration was determined by A260/A280 measurement. One microgram of total RNA was reverse transcribed into cDNA using AMV reverse transcriptase first strand cDNA synthesis kit according to the manufacturer's protocol (Marligen Biosciences, Sydney, Australia). Real-Time PCR was performed using a Bio-Rad IQ5 detection system, with reactions performed using SYBR Green Supermix (Bio-Rad, Sydney, Australia). Primers were designed using Primer 3, and obtained from GeneWorks (Hindmarsh, Australia; See Table 2). The amplification of cDNA samples was carried out using IQ SYBR green™ following the manufacturers protocols (BioRad, Sydney, Australia) Fluorescent emission data was captured and mRNA levels were analyzed using the critical threshold (CT) value [35]. Thermal cycling and fluorescence detection were conducted using the BioRad IQ5 sequence detection system (BioRad, Sydney, Australia). Samples were normalized for the cDNA concentration determined with OliGreen (Invitrogen, Mulgrave, Australia) [36].

### Protein & Enzyme Activity Analysis

Gastrocnemius muscle tissue samples (20 mg) were homogenized and centrifuged at 10,000 *g* at 4°C for 10 minutes. The protein concentration was determined via the Bradford method (BioRad, Sydney, Australia). Total SOD, glutathione peroxidase (GPx), catalase, and XO activity were measured with commercial enzyme assay kits as per the manufacturer's instructions (Sapphire Bioscience, Waterloo, Australia; Invitrogen, Mulgrave, Australia).

### Statistics

All statistical analyses were performed using SPSS Statistics Version 17.0 (IBM, Chicago, USA) or GraphPad Prism 5 (GraphPad Software, San Diego, USA), with results expressed as mean ± standard error of mean (SEM) and considered statistically significant if P<0.05, unless otherwise stated. Data was analyzed using two-way ANOVA, with a Tukey's test post hoc analysis performed to determine differences between groups where appropriate. Correlation was analysed using Pearsons Test Weight-loss data was analyzed using Repeated Measures ANOVA with Bonferroni post-hoc analysis.
